# Prevalence and Determinants Influencing the Use of Electronic Cigarette Smoking in Male Students of Taif University

**DOI:** 10.7759/cureus.40885

**Published:** 2023-06-24

**Authors:** Mhdee H Albgami, Ahmed S Alzahrani, Anwar M Alghamdi, Nawaf S Alamri, Ali H Alghamdi, Rami A Alsuwat

**Affiliations:** 1 Department of Preventive Medicine, Prince Mansour Military Hospital, Taif, SAU; 2 Department of Preventive Medicine, Armed Forces Hospital, Madina, SAU; 3 Department of Home Health Care, Prince Mohammed Bin Abdulaziz Hospital, Riyadh, SAU; 4 Department of ​Aviation Medicine, Prince Sultan Military Hospital, Taif, SAU

**Keywords:** youth, experimentation, e-cigarettes, behavior, attitude

## Abstract

Background and aim: Electronic cigarette (e-cigarette) smoking is a significant public health problem in Saudi Arabia, particularly among youth who use it as an alternative to traditional cigarettes. This study aimed to evaluate the prevalence and beliefs towards e-cigarette use among Taif University students in Saudi Arabia.

Methods: A cross-sectional study was conducted among male Taif University students using a self-administered questionnaire. A sample of 319 students was selected through stratified sampling. The questionnaire included questions about socio-demographic characteristics, smoking history, awareness of e-cigarettes, prevalence of e-cigarette use, beliefs towards e-cigarettes, and reasons for e-cigarette use.

Results: The study revealed a high prevalence of e-cigarette use among Taif University students, with 40.1% of participants having used e-cigarettes at least once during their lifetime and 43.7% believing that e-cigarettes are less dangerous than traditional cigarettes. Participants studying sciences had 0.76 times the odds of believing that e-cigarettes help smokers to quit compared to participants studying literature. Compared to smokers, ex-smokers had an OR of 34.1 (p<0.001) and non-smokers had an OR of 35.9 (p<0.001) for experimentation of e-cigarettes. Smokers who had friends that tried e-cigarettes had an OR of 6.6 (p<0.001) for trying e-cigarettes, compared to smokers who did not have such friends.

Conclusion: The study found that 40.1% of participants have used e-cigarettes at least once during their lifetime with a significant proportion of participants unaware of the potential health hazards of e-cigarettes, and many believed that e-cigarettes are less dangerous than traditional cigarettes. These findings emphasize the need for targeted educational interventions to address misconceptions and promote awareness among university students.

## Introduction

Tobacco smoking is the largest avoidable threat that causes several diseases affecting mainly the heart and lungs [1]. Every year, around six million people die because of tobacco smoking-induced diseases, either due to direct effects or non-smokers being exposed to second-hand smoke [2]. The prevalence of traditional cigarette smoking among male university students in the Kingdom of Saudi Arabia (KSA) has been previously reported to range from 13% in central Saudi Arabia [3] to 30.4% in Majmaah [4]. Tobacco-related diseases have claimed over 7,000 Saudi lives, yet more than 20,000 children and adolescents, as well as over three million adults, continue to smoke tobacco daily [5].

Recently, electronic cigarettes (e-cigarettes) have been introduced to KSA with limited knowledge about their prevalence and pattern of utilization. In a recent study, the prevalence of e-cigarette use among health science university students was 27.7% [6]. E-cigarettes are electronic emit-vapor devices that emit nicotine in an aerosol form. They are manufactured from a battery, mouthpiece, an automatic or manual switch, a cartridge, and an atomizer with a heating element that contains a solution called “e-liquids” which contain propylene glycol or glycerol (or both) and can contain flavors and additives [7].

E-cigarettes have been reported to induce addiction [8], and there is evidence of an increased risk of prolonged exposure to vapors chemicals of e-cigarettes that contain toxic substances, including formaldehyde [9]. On the other hand, there is debate about whether e-cigarette use can replace traditional cigarettes [10,11]. In general, the impact of e-cigarettes on the health of human beings is still under investigation, as no prolonged studies have been implemented yet [12]. The availability of e-cigarettes with sweet flavors for the young population presents a great concern as it simulates smoking behavior as well as facilitates addiction to nicotine, which, over time, could lead to the use of traditional tobacco smoking [13,14].

Qanash et al. in 2019 investigated e-cigarette use for smoking cessation among 1007 health science college students in Jeddah. Results showed that 14.1% smoked traditional cigarettes, with 22% smoking half a pack per day. Meanwhile, 27.7% smoked e-cigarettes, with 20% doing so regularly. Of these e-cigarette users, 42.7% used them to quit smoking, and 56.7% succeeded. Factors that increased the likelihood of quitting smoking were thinking traditional smoking is more addictive than e-cigarettes, using fruit-flavored e-cigarettes, or starting vaping to quit smoking [6].

E-cigarette smoking is a significant public health problem in Saudi Arabia, particularly among youth who use it as an alternative to traditional cigarettes [2]. Some researchers believe e-cigarettes help smokers quit, while others believe they should be banned for lack of data on efficacy and safety [10,11]. Limited studies have been conducted in Saudi Arabia. This study aimed to evaluate the prevalence and beliefs towards e-cigarette use among Taif University students in Saudi Arabia. It is an important step toward understanding this emerging public health issue, and it will inform policymakers and public health officials.

## Materials and methods

Study settings

A cross-sectional design was selected for this study. This study was conducted in Taif City, Saudi Arabia, at Taif University with a population of 683,000 (2019 estimate). The university has “13” colleges for male students, including “four” health-related colleges, “three” humanities of education, “two” sharia and administration, and “four” science and engineering.

Target population

The target population for this cross-sectional study was male Taif University students from different colleges enrolled throughout the academic year 2021-2022 (n=16,082). The inclusion criteria were male Saudi adult students in Taif University who are ≥18 years old. Higher education students (master's and Ph.D. students), university staff, and visitors were excluded from the study. Females were excluded from the study due to logistical challenges in data collection and the inability to obtain their information, which could potentially compromise the randomization process.

Sample size

The sample size was calculated using Cochran’s formula for estimating the sample size equation with a 95% confidence level (CI), 0.05 critical value, and 28% prevalence rate [15]. The calculated sample size was 310 male students. However, the sample was increased by approximately 10% to compensate for possible non-response or incomplete responses, making it 341 male students.

Sampling technique

A stratified sampling technique with proportional allocation was adopted. Stratification was done according to the type of colleges, whether scientific or literature. This resulted in two separate sampling frames, one for scientific colleges (n=7) and the other for literature colleges (n=5). One college was selected from each frame, and the sample was equally distributed between the two chosen colleges (170 students from each college). In colleges including branches, one or two branches were randomly selected by a simple random technique. Within each college, the sample was equally distributed between all academic levels. A simple random sampling technique was applied to select students from lists including names of students from the two lists of students.

Data collection tool

A self-administered questionnaire was utilized for data collection. It was composed of the following six main sections: socio-demographic characteristics of the students, such as age and academic level, smoking history, awareness of e-cigarettes (eight statements with yes/no responses), the prevalence of use of e-cigarettes (six statements with yes, neutral, and no responses), beliefs towards e-cigarettes (three statements with 5-point Likert scale response - totally agree, somewhat agree, neutral, somewhat disagree, totally disagree), and reasons for e-cigarette use. The questionnaire had been utilized previously in a study carried out in Romania among university students [16].

Permission to apply the questionnaire in the current study, with minimal modifications, was requested by the corresponding author through e-mail communication. The English language of the questionnaire was simple and could be easily understood by medical students. However, to ensure full understanding, it was translated and retranslated into Arabic language and validated by three family medicine consultants (face validity).

Data collection technique

The researcher visited Taif University (male section) after obtaining approvals. Permission from the university authority was taken. Self-administered questionnaires were distributed to selected students by their lecturers under the direct supervision of the researcher. Data collection was implemented during regular academic hours.

Ethical considerations

Written permission was obtained from the Joint Program of Preventive Medicine in Taif Region, as well as the deans of the involved colleges at Taif University. The researcher made efforts not to disturb the academic program of the students and arranged visits with the administration of each college in advance. Individual consent was obtained from each student before data collection. Acceptance to participate by filling out the questionnaire was considered sufficient consent. All information was kept confidential and was accessed only for the purpose of scientific research.

Statistical analysis

SPSS version 27.0 (Armonk, NY: IBM Corp.) was used for the data analysis. Frequencies and percentages were used for summarizing categorical variables. The dependent variable was coded to be binary. Subgroup analyses were done as suitable for each question. For which, we used binary logistic regression to calculate the odds ratios (ORs) with the independent variables. Significance was evaluated using the critical value (p<0.05).

## Results

The survey about prevalence and beliefs related to e-cigarettes was done among a total of 319 participants, including smokers, ex-smokers, and non-smokers. Among the participants, 50.2% were studying sciences, while 49.8% were studying literature. In terms of year, 24.5% were first-year students, 25.4% were second-year students, and similar proportions were in the third and fourth years. Regarding smoking status, 73.4% were non-smokers, 19.7% were smokers, and 6.9% were ex-smokers (Table 1).

**Table 1:** Academic level and smoking status of the students.

Variable	Groups	Count	n%
College	Sciences	160	50.20%
	Literature	159	49.80%
Year	First	78	24.50%
	Second	81	25.40%
	Third	80	25.10%
	Fouth	80	25.10%
Smoking	Non-smoker	234	73.40%
	Smoker	63	19.70%
	Ex-smoker	22	6.90%

The participants were asked to answer either "yes" or "no" to each question about e-cigarette awareness. The vast majority of participants (99.1%) have heard of e-cigarettes. However, awareness of specific facts about e-cigarettes varied, with 71.2% of participants aware that e-cigarettes are a nicotine delivery system, 56.4% aware that e-cigarettes are an appliance that vaporizes nicotine, and 54.5% aware that e-cigarettes can be inhaled with different additives like nicotine. A large majority of participants (94%) were aware that e-cigarettes can be inhaled with different flavors. However, only 28.2% of participants were aware that there is no combustion in an e-cigarette, and only 13.5% were aware that there is no carbon monoxide in an e-cigarette (Table 2). 

**Table 2:** Students’ responses to the awareness questions.

Question	Yes (%)	No (%)
Have you heard of electronic cigarettes (e-cigarettes)?	316 (99.1%)	3 (0.9%)
Are you aware that an e-cigarette is a nicotine delivery system?	227 (71.2%)	92 (28.8%)
Are you aware that an e-cigarette is an appliance that vaporizes nicotine?	180 (56.4%)	139 (43.6%)
Are you aware that an e-cigarette can be inhaled with different additives (i.e., nicotine)?	174 (54.5%)	145 (45.5%)
Are you aware that an e-cigarette can be inhaled with different flavors (i.e., peach)?	300 (94%)	19 (6%)
Are you aware that there is no combustion in an e-cigarette?	90 (28.2%)	229 (71.8%)
Are you aware that there is no carbon monoxide in an e-cigarette?	43 (13.5%)	276 (86.5%)

Three responses were included regarding the questions on e-cigarette use and attitudes "yes," "no," and "not sure." Of the total, 40.1% have used e-cigarettes at least once during their lifetime, while 18.8% have used them in the last month. Only 10.7% of participants intend to use e-cigarettes in the next year, while 44.8% have friends who have tried e-cigarettes. A small percentage (6.6%) have parents who have tried e-cigarettes, while a higher percentage (21.9%) have siblings who have tried e-cigarettes (Table 3).

**Table 3:** Response to prevalence and use of e-cigarettes among the students.

Question	Yes (%)	No (%)	Not sure (%)
I have used e-cigarettes at least once during my lifetime.	128 (40.1%)	13 (4.1%)	178 (55.8%)
I have used e-cigarettes in the last month.	60 (18.8%)	19 (6.0%)	240 (75.2%)
I intend to use e-cigarettes in the next year.	34 (10.7%)	59 (18.5%)	226 (70.8%)
I have friends who have tried e-cigarettes.	143 (44.8%)	60 (18.8%)	116 (36.4%)
I have parents who have tried e-cigarettes.	21 (6.6%)	37 (11.6%)	261 (81.8%)
I have siblings who have tried e-cigarettes.	70 (21.9%)	81 (25.4%)	168 (52.7%)

Further analyses among a total of 319 participants, including smokers, ex-smokers, and non-smokers were done and revealed that 43.7% of the participants agree that e-cigarettes are less dangerous than traditional cigarettes, while 32.9% do not know and 23.4% totally or partially disagree. Regarding the belief that e-cigarettes can help smokers to quit, 39.2% of participants totally or partially agree, while 31.3% do not know and 29.4% totally or partially disagree. Our results also showed that 40.5% of participants have used e-cigarettes at least once during their lifetime, while 19.0% have used them in the last month.

Among students who experimented with e-cigarettes, the survey found that 35.2% tried them because they believe e-cigarettes are less dangerous than traditional cigarettes, 25.8% to reduce the number of traditional cigarettes, 26.6% to quit smoking, 39.8% out of curiosity, and 37.5% because other friends also tried e-cigarettes. Regarding the intention to use e-cigarettes in the next year, the survey found that 10.8% of participants said yes, 18.7% were neutral, and 70.6% said no. Finally, the survey found that 45.3% of participants have friends who have tried e-cigarettes, 6.6% have parents who have tried e-cigarettes, and 22.2% have siblings who have tried e-cigarettes (Table 4).

**Table 4:** Awareness, behavior, and social influence relation with smoking experimentation related to smoking status. ٭The study sample consisted of students who have ever heard about e-cigarettes. ٭٭The percentages are calculated for students who ever tried e-cigarettes.

Beliefs		Total (319)	Smokers* (63)	Ex-smokers* (22)	Non-smokers (234)
E-cigarettes are less dangerous than traditional cigarette	I totally agree/I partially agree	43.7%	71.4%	63.6%	33.8%
	I do not know	32.9%	19.0%	13.6%	39.3%
	I totally disagree/I partially disagree	23.4%	9.5%	22.7%	26.9%
E-cigarettes can help smokers to quit	I totally agree/I partially agree	39.2%	74.6%	54.5%	27.8%
	I do not know	31.3%	7.9%	18.2%	39.7%
	I totally disagree/I partially disagree	29.4%	17.5%	27.3%	32.5%
E-cigarettes are used only by smokers	I totally agree/I partially agree	18.7%	11.1%	18.2%	20.5%
	I do not know	21.2%	11.1%	31.8%	23.9%
	I totally disagree/I partially disagree	60.1%	77.8%	50.0%	55.6%
Behavior	I have used e-cigarettes at least once during my lifetime	40.5%	100.0%	100.0%	21.8%
	I have used e-cigarettes in the last month	19.0%	64.9%	60.0%	4.7%
Reasons for trying e-cigarettes among students who experimented with them**	E-cigarettes are less dangerous	35.2%	38.1%	18.2%	42.1%
	To reduce the number of the traditional cigarettes	25.8%	41.3%	22.7%	45.6%
	To quit smoking	26.6%	39.7%	31.8%	43.9%
	Curiosity	39.8%	44.4%	18.2%	49.1%
	Other friends also tried e-cigarette	37.5%	44.4%	22.7%	49.1%
Intention to use e-cigarettes in the next year	Yes	10.8%	36.5%	31.8%	1.7%
	Neutral	18.7%	36.5%	27.3%	12.8%
	No	70.6%	27.0%	40.9%	85.5%
Social influence	I have friends who have tried e-cigarettes	45.3%	81.0%	63.6%	33.3%
	I have parents who have tried e-cigarettes	6.6%	3.2%	18.2%	6.4%
	I have siblings who have tried e-cigarettes	22.2%	38.1%	27.3%	17.1%

Further analysis was conducted to examine the relationship between college major, smoking status, beliefs about e-cigarettes, and the propensity for experimenting with e-cigarettes. The OR was reported wherever possible for all the investigated relations. Participants studying sciences had 0.76 times the odds of believing that e-cigarettes help smokers to quit compared to participants studying literature. Compared to smokers, ex-smokers had an OR of 34.1 (p<0.001) and non-smokers had an OR of 35.9 (p<0.001) for experimentation of e-cigarettes. Compared to smokers who did not intend to quit traditional smoking within the next year, those with the intention to quit had an OR of 4.1 (p=0.109) for experimenting with e-cigarettes. Smokers who had friends that tried e-cigarettes had an OR of 6.6 (p<0.001) for trying e-cigarettes, compared to smokers who did not have such friends. ORs for experimentation with e-cigarettes were also investigated among those having a parent or sibling who uses e-cigarettes (Table 5).

**Table 5:** Bivariate regression for odds ratios of e-cigarette experimentation among the total sample and current smokers.

Variables		Total sample (319)		Smokers (63)	
		OR	p-Value	OR	p-Value
College	Literature	1	0.235	1	0.967
	Sciences	0.76		1.04	
E-cigarettes help quitting smoking	I totally disagree/I partially disagree/I do not know	1	<0.001	1	0.957
	I totally agree/I partially agree	0.30		1.06	
E-cigarettes are less dangerous	I totally disagree/I partially disagree/I do not know	1	<0.001	1	0.061
	I totally agree/I partially agree	0.30		0.15	
E-cigarettes are used only by smokers	I totally disagree/I partially disagree/I do not know	1	0.036	1	0.004
	I totally agree/I partially agree	1.37		26.7	
Smoking behavior	Non-smoker	1	-	-	-
	Ex-smoker	35.9	<0.001	-	-
	Smoker	34.1	<0.001	-	-
Intention to quit traditional smoking within the next year	No	1	0.109	1	0.133
	Yes	4.1		3.75	
Friends tried e-cigarettes	No	1	<0.001	1	0.002
	Yes	6.6		35.7	
Parents tried e-cigarettes	No	1	0.041	-	-
	Yes	2.6		-	-
Siblings tried e-cigarettes	No	1	<0.001	1	0.532
	Yes	3.6		0.58	

## Discussion

E-cigarette use has increased significantly in recent years, particularly among young adults. The reason for its huge success is attributable to the misconception that e-cigarettes are safe compared to tobacco smoking [17]. The purpose of the present study was to determine the prevalence and determinants of e-cigarette smoking among university students. The present study revealed that 40.1% of the participants have used e-cigarettes at least once during their lives. These findings were higher than the previous study conducted in Saudi Arabia by Althobaiti and Mahfouz who reported that 26% of participants tried e-cigarettes once in their life. Similarly, a cross-sectional study by Qanash et al. reported that the prevalence of e-cigarettes was 27.7% among health science college students. The study included 1007 participants to determine the effectiveness of e-cigarettes on smoking cessation [6]. Their findings showed that more than half of the students (56.7%) who used e-cigarettes to quit smoking succeeded in doing so [6]. In the present study, 39.2% of participants agreed that e-cigarettes can help quit smoking whereas 26.6% of the participants used e-cigarettes for this purpose. A cross-sectional study by Awan also reported that 24.3% of his study participants used e-cigarettes for smoking cessation [18]. However, a much higher number of participants (58.7%) of Polish students reported the use of e-cigarettes to quit smoking [19]. Our findings were comparable to a study among American health professional students which reported 24.2% of patients used e-cigarettes at least once and among those 23.1% used for smoking cessation [20]. Apart from smoking cessation, several other determinants have also been described in the literature. A systematic review by Khanagar et al. determined the pattern of use and perception of the Saudi population toward e-cigarette smoking. The authors concluded that the usage of e-cigarettes among the Saudi population has several reasons including the intention to reduce tobacco smoking, considering e-cigarette as less harmful, less addictive, curiosity, lower cost, and influence of others [5].

In the present study, 43.7% of the participants totally or partially agreed that e-cigarettes are less dangerous than traditional cigarettes. Previously, several studies have shown that e-cigarettes do not demonstrate discernible immediate impacts on the physiological functioning of the cardiovascular and respiratory systems [5,21]. However, new evidence does not support the notion that e-cigarettes are safe as these have been demonstrated to increase respiratory flow resistance. E-cigarettes also decrease fractional exhaled nitric oxide as observed in conventional smoking [22]. Therefore, Word Health Organization has regarded the use of e-cigarettes as toxic [23]. A recent study by Reidel et al. reported that e-cigarettes alter the immune response in the lungs [24]. Several international studies have also investigated to identify the determinants of e-cigarette smoking. A multicentric cross-sectional study by Brożek et al. in Central and Eastern Europe investigated traditional cigarette and e-cigarette smoking among 14,352 university students and specified the factors associated with their smoking [25]. The overall prevalence of traditional smoking, e-cigarette smoking, and dual smoking were 12.3%, 1.1%, and 1.8%, respectively. Males were more likely to smoke both traditional cigarettes and e-cigarettes compared to females. There was a statistically significant difference between smokers and non-smokers regarding their perception of e-cigarettes, p<0.001 [25]. The present study only investigated the e-cigarette smoking behavior among male students which is the limitation of the study. Male gender has been identified as a significant factor associated with e-cigarette smoking. A cross-sectional study among university students in Malaysia also reported that a factor associated significantly with the exclusive use of e-cigarettes was the male gender [26].

The prevalence of e-cigarette smoking was 74.9%; 40.3% used both traditional and e-cigarettes (dual users), and 34.5% were exclusive e-cigarette users. Reported adverse effects of e-cigarettes were dizziness (14.4%), cough (14.1%), and headaches (12.4%). More than half (57.8%) of the users utilized e-cigarettes as a smoking cessation tool, while others considered e-cigarettes self or as part of social activities [26]. Similarly, in the present study, 39.8% of the participants tried e-cigarettes out of curiosity, and 37.5% because other friends also tried e-cigarettes. Similarly, a cross-sectional study by Lotrean in 2015 among 480 university students aged between 19 and 24 years reported that predictors of e-cigarette smoking were male gender, being a current smoker of traditional cigarettes, having a belief that e-cigarettes could help in quitting smoking, and having friends who tried e-cigarettes [27]. A study by Czoli et al. among young adults in Canada reported that current smokers were more likely to have tried e-cigarettes than ex-smokers (OR=2.3). The majority of smokers (80.4%) have tried e-cigarettes to help them quit smoking (80.4%) or to be the long-term replacement for cigarettes (77.8%), or to smoke them where they cannot smoke (80.9%) [28].

## Conclusions

The study revealed a high prevalence of e-cigarette use among Taif University students, with 40.1% of participants having used e-cigarettes at least once during their lifetime. A significant proportion of participants were unaware of the potential health hazards of e-cigarettes, and many believed that e-cigarettes are less dangerous than traditional cigarettes. The study highlights the need for public health campaigns and interventions to increase awareness of the risks associated with e-cigarette use and to correct misperceptions about their safety. To address this issue, policymakers should consider regulating the sale and marketing of e-cigarettes, and health educators should provide students with accurate information about the risks associated with e-cigarette use. Longitudinal studies should be conducted to assess the long-term effects of e-cigarette use on health outcomes, and further research should investigate the relationship between e-cigarette use and traditional cigarette smoking among university students in Taif.
